# Mixed-methods Assessment of Barriers to Physical Activity for Middle School Latina Girls

**DOI:** 10.4148/2572-1836.1154

**Published:** 2022-05

**Authors:** Marisol McDaniel, Ana Sofia Ocegueda, Daisy Morales-Campos, Deborah Parra-Medina

**Affiliations:** University of Texas at Austin; University of Texas at Austin; University of Texas at Austin; University of Texas at Austin

**Keywords:** physical activity, health disparities, neighborhood, mixed methods

## Abstract

Meeting physical activity guidelines is associated with positive mental, physical, and social health. However, most adolescent girls do not meet the recommended level of physical activity, with Latina girls even less likely than white girls to do so. Partnership for Girls (PG) sought to improve physical activity (PA) and reduce sedentary behaviors to prevent obesity and obesity-related health disparities among low-income Latina adolescent girls attending Westside San Antonio schools. This study utilizes mixed-methods data—qualitative formative assessments with parents of adolescent girls to gain further insight on the PA needs of Latina girls, and standard quantitative survey assessments to examine barriers and facilitators to PA from the perspective of the Latina girls. Results from the parent focus groups identify safety, cost, and neighborhood structure, such as traffic, as salient barriers to physical activity. In alignment with the focus groups, the feasibility study found that the middle school Latinas’ neighborhood environment was a major barrier to physical activity. Policymakers and planners must address the structural obstacles, such as neighborhood structure and safety concerns, to address physical activity disparities among Latina middle school girls.

## Introduction

Meeting physical activity guidelines is associated with positive mental, physical, and social health. However, most adolescent girls do not meet the recommended level of physical activity. Latina girls are even less likely than white girls to engage in the recommended level of physical activity. According to the Texas Youth Risk Behavior Study, in 2019, 21.6% of Latinas in high school were physically inactive, a total of at least 60 minutes on none of the past seven days compared to 15.7% of white girls in high school (Larsen et al., 2018). Latinas were also more likely to be physically active for a shorter duration than white peers (Armstrong et al., 2018). Also, magnifying the behavioral disparity, Latinas are at increased risk for chronic disease (i.e., obesity, diabetes, heart disease) associated with a sedentary lifestyle.

In addition to schools that provide middle and high school Latinas opportunities to engage in physical activity through organized sports and physical education classes, many interventions specifically target Latina and immigrant families, such as the Niñas Saludables Study for middle and high school girls. However, many ineffective interventions did not significantly change objective accelerometry outcomes but highlighted barriers to overcome through future iterations. For example, a photovoice project identified parental restrictions and work, transportation, and safety issues as barriers to afterschool physical activity programs for Latinas aged 14-19 years old (Hannay et al., 2013).

Self-efficacy is associated with time spent in physical activity for adolescent Hispanic girls. However, self-efficacy may be an essential correlate, not a predictor (Alert et al., 2019). Perceived social support is also related to PA, mediated through perceived barriers (Dishman et al., 2010). Parental physical activity support is associated with higher odds of engaging in five or more days of moderate to vigorous physical activity (MVPA; Benes et al., 2017). Peer support is also associated with moderate to vigorous activity in young Latinas (Benes et al., 2017). Conversely, gender-based teasing from peers heightens feelings of insecurity among Latina girls, making them less likely to participate in group sports (Lopez, 2018).

Transportation is also a pertinent barrier for Latina girls/adolescents (Kelly et al., 2010). Transportation difficulties dis-proportionately impact Spanish-speaking Hispanics. It may be challenging to drive to a different location to engage in physical activity when unable to find a ride to run essential errands (Careyva et al., 2018). Even when attempting to be physically active close to home, the physical neighborhood environment may deter physical activity, especially if uneven sidewalks, potholes, or stray dogs run rampant (Morales-Campos et al., 2015). In addition to physical barriers, such as transportation and neighborhood environment, many social factors influence sedentary behavior and physical activity. On the other hand, supportive neighborhoods and safe school environments are associated with Latinas engaging in physical activity (Benes et al., 2017).

This study is grounded in the Bronfenbrenner ecological systems theory, which explains that interactions across multiple levels of influence and intersectionality theory affect human development. Physical activity among Latina girls has many levels of influence, including sociodemographic characteristics and cost (individual-level), interactions with classmates and parents (interpersonal-level), school physical activity and sports programs (institutional-level), and neighborhood factors (community-level) (Bronfenbrenner, 1992). We hypothesize that physical activity is associated with barriers at multiple levels of influence.

By focusing on physical activity barriers and understanding the experiences of Latinas with multiple marginalized identities, we aim to identify and conceptualize the social inequities that shape their experience and identify opportunities for intervention that address said inequities to increase physical activity. This study also utilizes multiple triangulation perspectives, in this case the girl and parent, to understand the phenomena of barriers to physical activity for Latinas in middle school in San Antonio, TX (Padgett, 2016).

## Methods

Partnership for Girls (PG) centers on improving physical activity (PA) and reducing sedentary behaviors to prevent obesity and obesity-related health disparities among low-income Latina adolescent girls in Westside San Antonio through a community partnership with Girl Scouts. The project’s goal was to design, implement, and evaluate a sustainable, peer-oriented, remote-technology intervention to increase PA and reduce sedentary behaviors among girls with the support and insight of the local community (Be Fit with Friends or BFF). Mixed-method approaches were used to achieve this goal. First, formative assessments were conducted to better understand the PA issues and concerns from the perspectives of community stakeholders, parents, and girls. Second, community stakeholders participated in in-depth individual interviews to share their views on the PA needs of girls. Focus groups were performed with parents of adolescent girls to gain further insight regarding the benefits of and barriers to PA in their community. Fourth, girls participated in a photovoice project that informed researchers on the environmental barriers to PA and a media survey that characterized the media behaviors of girls (Morales-Campos et al., 2015). Altogether, these assessments help inform the development of the BFF intervention. During the last phase of the study, BFF was tested twice, in a feasibility study and pilot study, to assess its feasibility, cultural appropriateness, and preliminary effectiveness. Girls completed pre-and post-test (6 months later) standardized assessments to examine barriers and facilitators to PA, change in PA and sedentary behaviors, and anthropometrics. Self-reported data were recorded using PDAs with sound-enhanced data collection. As part of the study, parents completed a brief demographic questionnaire and a few questions about their daughter’s PA. All participants received an incentive (e.g., Girl Scouts accessories) for completing the assessments. This study used results from the parent focus group study and baseline data from the feasibility and pilot studies.

The project was a collaborative partnership among the University of Texas Health Science Center at San Antonio’s (UTHSCSA) Institute for Health Promotion Research (IHPR), The University of Texas at San Antonio (UTSA), Girl Scouts of Southwest Texas (GSSWT), and the Edgewood Family Network (EFN). The Institutional Review Board approved the study procedures of the University of Texas Health Science Center at San Antonio (UTHSCSA). GSSWT was the primary community research partner, all partners aligned on goals to promote girls’ health and increase opportunities for physical activity. The GSSWT empowerment and leadership approach (Look, Think, Act) aligned with the research team’s CBPR approach, including engagement of all stakeholders in program planning, including the girls.

### Setting

The study took place in Westside San Antonio, Texas, a historically underserved urban sector of Latinos. The parents’ focus group study took place between January and April of 2010. Promotoras (community health workers) identified focus group participants (*n* = 31) who were parents of Hispanic/Latino heritage with a daughter in middle school through existing networks in the community. Promotoras prescreened potential subjects over the telephone or in person to determine their initial eligibility and interest in a study. A research assistant provided each focus group member with a study information sheet during the planned focus group. We did not require a signature from the subjects documenting consent for focus group participation. Participants had the opportunity to ask any questions before completing the demographic questionnaire and beginning the focus group.

The feasibility study recruited participants (girls and parents) from eight Girl Scout troops in Westside San Antonio (*n* = 39). All recruitment was completed by the Girl Scouts. Baseline data collection for the feasibility study took place in the Fall of 2010. Recruitment goals were challenging for various reasons (i.e., few troops existed in the target area, and there were several internal transitions in leadership and staffing within Girl Scouts). However, the reality of recruitment challenges in the feasibility study resulted in the collaborative development of a modified recruitment strategy. For the pilot study, promotoras recruited girls and parents from two public middle schools (*n* = 49), an afterschool program, and a physical education class, as well as from a community-based sample of girls from the local community who were not involved in Girl Scouts or who attended the middle schools (*n* = 61). We do not think this introduced selection bias because the girls were recruited from the community for the study, and also received a free GSSWT membership; the girls did not have to be a Girl Scout member previously. The pilot study was conducted in Fall 2011.

### Participants

#### Focus group study.

The participants were parents or primary caregivers (*n* = 31) of Latina girls between 11 and 14 years old who resided in Westside San Antonio. Participants were recruited by promotoras (community health workers) from the Edgewood Family Network by word of mouth and flyers placed throughout the community. The focus groups (*n* = 4; 2 female groups and 2 male groups) were conducted at the Edgewood Family Network program site at an elementary school not used by the district, located in Westside San Antonio. Participants received a $30 gift card incentive. Focus group participants were majority female; 58.6% had less than a high school education or equivalent (GED); two-thirds were born in Mexico, and had lived in the United States for an average of 18 years (see Table 1). In addition, female participants were on average younger (*M* = 39.4, *SD* = 7.1) than males (*M* = 48.6 years, *SD* = 16.4).

#### Feasibility and pilot studies.

The feasibility (*n* = 39) and pilot studies (*n* = 110) recruited Latina girls between 11 and 14 years old and their parents residing in Westside San Antonio, Texas. Parent participants in the pilot and feasibility studies were independent of parents that participated in the focus groups. The overwhelming majority of girls in both studies were born in the United States (over 90%); over half of their parents were U.S.-born (see Table 1). Approximately two-thirds came from two-parent households with household incomes below $30,000 per year. On average, there were two children in the family.

### Measurement

#### Focus group protocol.

To understand the parents’ perspective on physical activity among Latina girls, focus groups were conducted with two groups of fathers and two groups of mothers. Parents were asked about health knowledge, their daughters’ typical physical activity, neighborhood environment, and their input in creating a new physical activity program. These focus groups lead to conversations about facilitators and barriers to physical activity, especially in an urban area.

#### Physical activity barriers reported by girls.

As the outcomes of interest, we assessed three constructs related to potential PA barriers girls may experience: self-efficacy, social support, and perceived environment supporting physical activity. First, the eight-item Self-Efficacy for Physical Activity Scale (Saunders et al., 1997) measured the girls’ confidence in their ability to engage in PA (e.g., “I can be physically active during my free time on most days;” 1 = Disagree a lot to 5 = Agree a lot). A mean score was calculated, where higher scores were suggestive of better self-efficacy (alpha = .78). Second, the 13-item Social Support and Social Influence for Physical Activity Scale (Sallis et al., 1998; Sallis et al., 1987) measured the extent of children’s social support from their family and friends to engage in PA (1 = None to 5 = Very Often) (Saunders et al., 1997; Trost et al., 1999). A mean score was calculated, where higher scores were indicative of greater social support (alpha = .90). Lastly, the Perceived Environmental Support for Physical Activity Questionnaire examined perceptions children have about the physical and social environments at home and in their neighborhood that facilitate or impede PA (0 = No, 1 = Yes; (Hume et al., 2006)). Environmental perceptions were assessed in the following contexts: (1) PA opportunities at home, (2) neighborhood physical environment, (3) neighborhood aesthetics, (4) neighborhood safety, and (5) social environment. For the PA opportunities at home subscale (16 items), girls indicated whether there were certain items available at home that facilitated PA (e.g., trampoline, swimming pool) (Evenson et al., 2007). A summed score specified the total number of PA opportunities at home (range 0-16). Perceptions of the physical environment (12 items) examined whether girls walked or rode their bikes to several neighborhood destinations (e.g., their friends’ houses, the mailbox) (Mota et al., 2007). A total score was calculated to indicate the number of accessible neighborhood destinations (range 0-12). Girls also provided information about the aesthetics of their neighborhood (5 items, e.g., lots of nice houses, nice gardens) and safety (8 items, e.g., feeling safe crossing the road, heavy traffic). Negatively worded items were reverse coded so that a response in the affirmative was suggestive of better aesthetics and safety. Two separate scores were calculated for neighborhood aesthetics (range 0-5) and safety (range 0-8). Seven items assessed the perceived social environment (e.g., having friends in their area, having other children living next door). Higher scores suggest more positive social environments (range 0-7).

### Statistical Methods

Focus groups were audio-recorded, transcribed verbatim, and analyzed using Atlas.ti (7.0) in the source language. DMC and SO analyzed the transcripts using the grounded theory approach (Chun Tie et al., 2019; Glaser & Strauss, 1967; Hill et al., 2005; Strauss & Corbin, 1990). Both coders independently read the transcripts and then coded one transcript to identify emerging themes related to barriers at the individual, interpersonal, and environmental levels and facilitators promoting physical activity. Next, both discussed the original “coding list” and refined the list together. Finally, DMC and SO coded the remaining transcripts together using ATLAS.ti (7.0), meeting biweekly with the research team to discuss and refine the “coding list” of themes found in subsequent transcripts. We resolved discrepancies by reviewing and discussing the data until we reached a consensus.

Descriptive analysis and calculation of scores, including means and percentages for the focus group and feasibility study, were completed using the quantitative data collected during the feasibility and pilot studies. All quantitative analysis was completed using STATA (StataCorp, 2021).

## Results

### Parent-informed Physical Activity Barriers

#### Individual Barriers

##### Sedentary behaviors.

The young Latinas’ sedentary behaviors were mentioned three times in the focus group as barriers to physical activity (Table 3). Some parents discussed their daughters not engaging in PA due to increased generational use of technology or grooming behaviors. For example, one mother said, “Well them being inside the house can be the reason, because now more than ever they entertain themselves with the phones, doing their hair, watching television…” Another father had a similar insight, saying that “the major distraction they can have is the television.”

#### Interpersonal Barriers

##### Lack of time.

Due to the need to work long hours to provide for their families, some parents expressed that lack of time served as a barrier for their daughters doing physical activity. One father said that “working 12 hours from 5 [a.m.] to 5:30 [p.m.] is very hard.” Another parent mentioned that both parents might need to work, so the time they could dedicate toward their daughters’ physical activity is minimal. In addition to long work hours, others expressed their daughters had limited time due to their large school workload. One father stated that his kids “have to finish their homework before going outside and when they are finished, it is already dark, and they can’t go out.”

##### Parental role.

The topic of parental role, responsibilities, and supervision came up for a few parents when discussing barriers to physical activity. Meeting their kids’ needs and setting a good example was of value to some parents, but not reaching this need to their full potential could bring barriers. For example, one mother explained, “If I have depression…and I don’t take them, I am, in part, like a guilty mother so they have the inconvenience of not having physical activity in that way.”

#### Environmental Barriers

##### Affordability.

The parents in the focus groups mentioned cost or affordability a total of 5 times during the focus groups, and this was usually mentioned by the dads. Parents were asked by their daughters to pay for membership to gyms in the area, but the upfront cost and monthly membership were a disincentive, “I had to pay 90 and 45 ahead of time I told him, hear me out, they charge ahead of time like cable.”

##### Lack of safety.

One of the biggest concerns both mothers and fathers expressed was the lack of safety in their neighborhood, which prevents their daughters from going outside. A father described concerns related to traffic, saying, “Saturdays there’s no way for the girls to be there playing with their bicycles because they pass through here like devils.” A mother also described worries surrounding gun violence, expressing that their house had been hit before. As a result, she says, “one has the fear that one day one of the bullets will hit here, with one of the girls.”

##### Crime.

In addition to lack of safety, parents described criminal activity in their neighborhood as being an additional barrier to physical activity. One mother told of a conversation with her daughter about not letting her go anywhere, especially knowing that her daughter was aware of the crime in the neighborhood. She said, “a car came, someone got out, and they got in another, and the same car came, and they did a drug exchange…she says, ‘you never let me do anything because you always want me inside,’ I tell her well, it’s because of that same reason.” A father also described concerns with men in the neighborhood, saying, “I tell my wife to not let the girls go on their bikes over there because a man was talking to the women that would walk by, he would call them over to his house.”

##### Neighborhood.

Parents mentioned several neighborhood factors presenting barriers to their daughters’ physical health, one of the most salient themes, over 11 times. High amounts of traffic, loose dogs, sexual predation, and lack of other kids in the neighborhood were mentioned as reasons that served as barriers for their daughters. One mother said, “My girl doesn’t go out because my neighbors don’t have children; on the whole block, she just doesn’t have anyone to play with.” Contrarily, parents also mentioned community resources that provided dependable access to physical activity, such as Folklorico classes offered at school and park programs.

### Feasibility and Pilot Study Findings

The feasibility and pilot studies paralleled the focus group findings. For self-efficacy, the mean score was 3.94, as many girls agreed to their confidence in their ability to engage in PA. The social support for physical activity measured both family and friend support; the girls in the feasibility and pilot studies marginally scored higher for friend support, with important people in their life sometimes providing social support. Finally, the most notable result from the feasibility and pilot study was the perceived environmental support for physical activity. Neighborhood environment, measured by whether the girls travel via bike or walk around the neighborhood, averaged at the bottom 25% of the scale, suggesting neighborhood environments dangerously not conducive to physical activity. The atmosphere within the home was also not conducive to physical activity, with most participants scoring home opportunities low. The social environment score was also low, signifying that not many friends or children live close to the girl respondents with whom to engage in physical activity alongside.

## Discussion

Through Bronfenbrenner’s socio-ecological lens, barriers existed at each level: individual, interpersonal, and environmental, though this study identified environmental barriers as the most striking. For example, Latina girls and their parents reported similar barriers to physical activity, specifically environmental barriers such as neighborhood structure. In addition, the parents’ qualitative findings concerning fear of exposure to criminal activity provided greater evidence as to why the girls perceive a lack of social environment. For example, the girls may not have friends in the neighborhood because they cannot interact with other kids in the community. Another notable environmental barrier not mentioned by the parents but evident for the girls was the lack of home opportunities for PA; the girls felt like the home environment was not conducive to physical activity.

From an implementation science perspective, physical activity promotion interventions for Latina girls should address environmental barriers to physical activity, such as providing a space to foster community cohesion to facilitate the PA social environment. For example, if the parents know other parents close to them, they may be more willing to have their children play outside together. Many recent frameworks have integrated equity measures into their design—specifically, the concept of cost or burden (Shelton et al., 2020). Cost can include the overall burden to the community, the cost of time for individuals who may be working multiple jobs and juggling various responsibilities, the cost of transportation, and rehashing historical trauma (Eslava-Schmalbach et al., 2019). The barriers to physical activity among middle school Latina girls presented in this study are barriers that could be quickly addressed with intervention planning and equitable policies. Researchers and practitioners can address the lack of time by providing convenient opportunities for middle school Latina girls to engage in physical activity. For example, small physical activity sequences can be integrated into class time to increase attention and focus and move muscles (Alvidrez et al., 2019).

The lack of opportunities for PA within the home for middle school Latinas should also be deliberately explored further in public health research. For example, is a lack of physical activity opportunities because of a lack of space or time? How feasible would it be to conduct an intervention testing whether having a portable trampoline in the house increased home opportunities for PA? However, if researchers are interested in testing the environment, interventions must also address the sedentary behaviors mentioned by the study’s parents.

This study provides a pointed look at the barriers Latina middle schoolers face, a noted strength of this study from the perspective of both the girls and parents. The main limitation of the study is that the parents in the focus groups were not necessarily the parents of the daughters who participated in the feasibility and pilot studies. The sample sizes were also small. However, the results from this study can inform policy, PA, and other healthy lifestyle interventions targeting young Latinas; the barriers to physical activity are multi-level and multi-faceted. Many of the findings regarding environmental barriers to physical activity would remain consistent over time. In the context of the COVID-19 pandemic, middle school girls may have even less opportunity to participate in physical activity after school. If we were to replicate data collection efforts now, we would capture data via online surveys and paper, and focus groups would be held in-person and remotely. Findings may be different due to remote data collection, primarily if sessions outside of working hours are available, and more parents would be able to participate. Intervention to decrease the obstacles to PA must address the intertwined realities and concerns involving safety, the PA social environment, and the neighborhood environment.

## Figures and Tables

**Table 1:** Sample Characteristics of Parents in the Focus Group Study and Girls in the Feasibility and Pilot Studies; Partnership for Girls (2010 – 2011)

	Focus Group Mothers (*n* = 16)	Focus Group Fathers (*n* = 15)	Feasibility and Pilot Study Samples (*n* = 145)
	Mean (SD) or %	Mean (SD) or %	Mean (SD) or %
*Parents*			
Age	39.4 (7.1)	48.6 (16.4)	38.94 (7.36)
Male	0.0%	100%	6.98%
U.S. born	31.3%	60%	59.31%
At least one parent employed full-or part-time			60.14%
Household income $30k or more			32.17%
Marital status			
Never married	0.0%	0%	12.41%
Married/cohabitating	81.3%	100%	64.13%
Divorced/separated/widowed	18.8%	0%	23.46%
Number of adults in household			2.07 (0.84)
Number of children in household			2.58 (1.19)
			
*Girls*	*Focus Group*		*Feasibility Survey*
U.S. Born	31.3%		91.78%
*Parent Physical Activity Outcomes*			
Child stays inside afterschool			57.93%
Days per week child stays inside^a^			4.72 (1.31)

*Note.* Shaded cells indicate data for that variable were not collected or that data measures were not uniform across the feasibility and focus groups.

**Table 2:** Salient Themes from the Parents in the Focus Group; Partnership for Girls (2010-2011) (n = 21)

Themes	Saliency (mentions by whom)	Selected Quote
**Individual**		
Sedentary behaviors	3 (mother and fathers)	**“Yo digo que la mayor distracción que pueden tener es la televisión, o sea, tienen mucho tiempo viendo televisión, ¿verdad? O sea ¿verdad?”** “I say that the greatest distraction that they can have is the television, I mean, they have too much time watching television, right? I mean, right?”
**Interpersonal**		
Time	3 (mothers and fathers)	**“Tiempo del papá y la mamá porque tienen en que trabajar los dos, uno, la otra, es que antes en la escuela iban a aprender y a la casa llevaban algo de tarea…”** “The father’s time and the mother’s because both have to work, one, the other is that before in school, they went to learn, and they brought some homework to the house.”
Parental role	9 (mothers and fathers)	**“no querían que fueran los parientes que porque se distraen, dije no, yo tengo que mirar lo que hacen.”** “They didn’t want the parents to go [into the classroom OR watch] because they [the child] would get distracted; I said no, I have to watch what they do.”
**Environmental**		
Affordability	5 (majority fathers)	**“Ahí, cerca de la casa hay una como, no es mi academia, pero es chiquita, pero cobran 90 dolares de inscripción y 45 por mes, una nieta mía quería, le digo, no hombre.”** “There, close to the house there is like a, it is not my school, but it’s small, but they charge 90 dollars to sign up and 45 dollars a month, one of my nieces wanted to, I told her, no way.”
Crime	2 (mothers and fathers)	**“Pues es que uno protege a sus hijos, pero nada más brincando las niñas ya saben el movimiento de lo que traen de drogas y todo eso porque yo vivo en un four way stop”** “Well it’s because we protect our kids, but the girls simply jumping (outside) and they know the movement of drugs and all that because I live at a four way stop…”
Safety	6 (mothers and fathers)	**“Los sábados no tiene ni para que anden las niñas ahí jugando con las bicicletas porque pasan como diablos.”** “On Saturdays it doesn’t even have so that the girls can be playing with the bikes because they are passing like devils.”
Neighborhood	11 (majority mothers)	**“A mi niña le gusta jugar al half court pero cuando vamos al Woodlawn, hay puros mayores jugando, y no hay chanza, ya cuando ellos se van ya es tarde.”** “My daughter likes to play half court but when we go to Woodlawn, there are only older kids playing, and there is no chance, when they leave it is already late.”
Community resource	12 (mothers and fathers)	**“Pues en el parque hay el programa de la ciudad, junto con los demás parques y es donde van mis niños.”** “Well at the park there is a city program, along with the other parks and it is where my kids go.”

**Table 3:** Physical Activity Outcomes of Girls in the Feasibility and Pilot Studies, Partnership for Girls (2010-2011) (n = 145)

Girls’ Physical Activity Outcomes	Range	Mean (SD)
**PA Self-efficacy**	1 (Disagree a lot) – 5 (Agree a lot)	3.94 (0.71)
**PA Social Support**	1 (None) – 5 (Very often)	
Family Support		3.06 (0.87)
Friend Support		3.10 (0.96)
**PA Perceived Environment**		
Home Opportunities	0 (None) – 16 (Maximum accessible)	4.91 (2.87)
Neighborhood	0 (None) – 12 (Maximum accessible)	2.53 (2.25)
**Environment**		
Aesthetics	0 (None) – 5 (Maximum score for 5 items)	3.40 (1.28)
Safety	0 (None) – 8 (Maximum score for 8 items)	5.12 (1.96)
Social Environment	0 (None) – 7 (Maximum score for 7 items)	3.23 (1.97)

*Note.* Higher number depicting positive outcome; specifics in Focus Group Protocol section

## References

[R1] Alert MD, Saab PG, Llabre MM, & McCalla JR (2019). Are self-efficacy and weight perception associated with physical activity and sedentary behavior in Hispanic adolescents? Health Education & Behavior, 46(1), 53–62. 10.1177/1090198118788599 30117340

[R2] Alvidrez J, Castille D, Laude-Sharp M, Rosario A, & Tabor D (2019). The National Institute on Minority Health and Health Disparities Research Framework. American Journal of Public Health, 109(S1), S16–S20. 10.2105/ajph.2018.304883 30699025 PMC6356129

[R3] Armstrong S, Wong CA, Perrin E, Page S, Sibley L, & Skinner A (2018). Association of physical activity with income, race/ethnicity, and sex among adolescents and young adults in the United States: Findings from the National Health and Nutrition Examination Survey, 2007-2016. JAMA Pediatrics, 172(8), 732–740. 10.1001/jamapediatrics.2018.1273 29889945 PMC6142913

[R4] Benes D, Dowling J, Crawford S, & Hayman LL (2017). Social and environmental influences on physical activity levels in Latina adolescents. Public Health Nursing, 34(2), 101–111. 10.1111/phn.12278 27384961

[R5] Bronfenbrenner U. (1992). Ecological systems theory. Jessica Kingsley Publishers.

[R6] Careyva BA, Hamadani R, Friel T, & Coyne CA (2018). A social needs assessment tool for an urban Latino population. Journal of Community Health, 43(1), 137–145. 10.1007/s10900-017-0396-6 28707180

[R7] Chun Tie Y, Birks M, & Francis K (2019). Grounded theory research: A design framework for novice researchers. SAGE Open Medicine, 7, 2050312118822927. 10.1177/2050312118822927 PMC6318722 30637106

[R8] Dishman RK, Dunn AL, Sallis JF, Vandenberg RJ, & Pratt CA (2010). Social-cognitive correlates of physical activity in a multi-ethnic cohort of middle-school girls: Two-year prospective study. Journal of Pediatric Psychology, 35(2), 188–198. 10.1093/jpepsy/jsp042 19468040 PMC2902830

[R9] Eslava-Schmalbach J, Garzón-Orjuela N, Elias V, Reveiz L, Tran N, & Langlois EV (2019). Conceptual framework of equity-focused implementtation research for health programs (EquIR). International Journal for Equity in Health, 18(1), Article 80. 10.1186/s12939-019-0984-4 31151452 PMC6544990

[R10] Evenson KR, Scott MM, Cohen DA, & Voorhees CC (2007). Girls’ perception of neighborhood factors on physical activity, sedentary behavior, and BMI. Obesity, 15(2), 430–445. 10.1038/oby.2007.502 17299117

[R11] Glaser BG, & Strauss AL (1967). The discovery of grounded theory: Strategy for qualitative research. Aldine Transaction.

[R12] Hannay J, Dudley R, Milan S, & Leibovitz PK (2013). Combining photovoice and focus groups: Engaging Latina teens in community assessment. American Journal of Preventive Medicine, 44(3, Supplement 3), S215–S224. 10.1016/j.amepre.2012.11.011 23415186

[R13] Hill CE, Knox S, Thompson BJ, Williams EN, Hess SA, & Ladany N (2005). Consensual qualitative research: An update. Journal of Counseling Psychology, 52(2), 196–205. 10.1037/0022-0167.52.2.196

[R14] Hume C, Ball K, & Salmon J (2006). Development and reliability of a self-report questionnaire to examine children’s perceptions of the physical activity environment at home and in the neighbourhood. International Journal of Behavioral Nutrition and Physical Activity, 3(1), Article 16. 10.1186/1479-5868-3-16 16846519 PMC1550247

[R15] Kelly EB, Parra-Medina D, Pfeiffer KA, Dowda M, Conway TL, Webber LS, Jobe JB, Going S, Pate RR (2010). Correlates of physical activity in black, Hispanic, and white middle school girls. Journal of Physical Activity & Health, 7(2), 184–193. 10.1123/jpah.7.2.184 20484757 PMC2937261

[R16] Larsen B, Benitez T, Cano M, Dunsiger SS, Marcus BH, Mendoza-Vasconez A, Sallis J, & Zive M (2018). Web-based physical activity intervention for Latina adolescents: Feasibility, acceptability, and potential efficacy of the Niñas Saludables study. Journal of Medical Internet Research, 20(5), e170. 10.2196/jmir.9206 29743151 PMC5966649

[R17] Lopez V. (2018). No Latina girls allowed: Gender-based teasing within school sports and physical activity contexts. Youth & Society, 51(3), 377–393. 10.1177/0044118X18767772

[R18] Morales-Campos DY, Parra-Medina D, & Esparza LA (2015). Picture this!: Using participatory photo mapping with Hispanic girls. Family & Community Health, 38(1), 44–54. 10.1097/fch.0000000000000059 25423243 PMC4244704

[R19] Mota J, Gomes H, Almeida M, Ribeiro JC, Carvalho J, & Santos MP (2007). Active versus passive transportation to school–differences in screen time, socio-economic position, and perceived environmental characteristics in adolescent girls. Annals of Human Biology, 34(3), 273–282. 10.1080/03014460701308615 17612859

[R20] Padgett DK (2016). Qualitative methods in social work research (Vol. 36). Sage Publications.

[R21] Sallis JF, Bauman A, & Pratt M (1998). Environmental and policy interventions to promote physical activity. American Journal of Preventive Medicine, 15(4), 379–397. 10.1016/S0749-3797(98)00076-2 9838979

[R22] Sallis JF, Grossman RM, Pinski RB, Patterson TL, & Nader PR (1987). The development of scales to measure social support for diet and exercise behaviors. Preventive Medicine, 16(6), 825–836. 10.1016/0091-7435(87)90022-3 3432232

[R23] Saunders RP, Pate RR, Felton G, Dowda M, Weinrich MC, Ward DS, Parsons MA, & Baranowski T (1997). Development of questionnaires to measure psychosocial influences on children’s physical activity. Preventive Medicine, 26(2), 241–247. 10.1006/pmed.1996.0134 9085394

[R24] Shelton RC, Chambers DA, & Glasgow RE (2020). An extension of RE-AIM to enhance sustainability: Addressing dynamic context and promoting health equity over time. Frontiers in Public Health, 8, Article 134. 32478025 10.3389/fpubh.2020.00134 PMC7235159

[R25] StataCorp. (2021). Stata Statistical Software: Release 17. In StataCorp LLC.

[R26] Strauss A, & Corbin J (1990). Basics of qualitative research: Grounded theory procedures and techniques. Sage publications.

[R27] Trost SG, Pate RR, Ward DS, Saunders R, & Riner W (1999). Correlates of objectively measured physical activity in preadolescent youth. American Journal of Preventive Medicine, 17(2), 120–126. 10.1016/s0749-3797(99)00056-2 10490054

